# Supplementary material for: Psychotherapeutic Interventions to Improve Psychological Adjustment in Type 1 Diabetes: A Systematic Review
Source: Int J Environ Res Public Health. 2021 Oct 18;18(20):10940. doi: 10.3390/ijerph182010940 (PMC8535719; doi:10.3390/ijerph182010940)
Supplement: Supplementary file 1 [file ijerph-18-10940-s001.zip › ijerph-1373390-supplementary.pdf]

Supplementary

Table S1. Search strategy for Ovid MedLine.

	**Identifies interventions TI, AB**
1	
2	
3	
4	
5	
6	
7	1 or 2 or 3 or 4 or 5 or 6
	Identifies type 1 diabetes mellitus TI, AB
8	
9	
10	
11	
12	
13	
14	
15	
16	
17	
18	
19	
20	
21	
22	
23	8 or 9 or 10 or 11 or 12 or 13 or 14 or 15 or 16 or 17 or 18 or 19 or 20 or 21 or 22
	Identifies psychological wellbeing TI, AB
24	
25	
26	
27	
28	
29	
30	
31	
32	
33	
34	24 or 25 or 26 or 27 or 28 or 29 or 30 or 31 or 32 or 33
35	7 and 23 and 35

Table S2. a. Study quality, risk of bias in RCT.

Study	Randomization process	Deviations from intended interventions	Mising outcome data	Measurement of the outcome	Selection of the reported result	Overall Bias
Van der ven, et al. (2005)	Some concerns	Some concerns	Low	Some concerns	Some concerns	Some concerns
Serlachius, et al. (2014)	Some concerns	Some concerns	Low	High	Low	High
Menting, et al. (2018)	High	Some concerns	Low	High	High	High
Fisher, et al. (2018)	High	Some concerns	Low	High	Some concerns	High
Elllis, et al. (2005)	Some concerns	Some concerns	Low	High	Some concerns	High
Channon, et al (2007)	Some concerns	Some concerns	Low	Some concerns	Some concerns	Some concerns
Ambsberg, et al. (2009)	Some concerns	Low	Low	High	Some concerns	High

Table S3. b. Study quality, risk of bias in cohort study.

**Study**	**Selection**				Comparibility	Outcome		
	**Representativeness of the exposed cohort**	**Selection of the non-exposed cohort**	**Ascertainment of exposure**	**Outcome was not present at start of study**	Study controls for gender/study controls for any additional factor	Assessment of outcome	Was follow-up long enough for outcomes to occur[a]	Adequacy of follow-up of cohorts
Forlani et al., 2013	*	*			*/		*	*
